# A Double-Blind Randomized Clinical Trial for Evaluation of Galactogogue Activity of *Asparagus racemosus* Willd.

**Published:** 2011

**Authors:** Mradu Gupta, Badri Shaw

**Affiliations:** a *Department of Dravyaguna****, ****Institute of Post Graduate Ayurvedic Education and Research, 294/3/1, A.P.C. Road, Kolkata 700009.*; b *Department of Kayachikitsa****, ****Institute of Post Graduate Ayurvedic Education and Research, 294/3/1, A.P.C. Road, Kolkata 700009 , India.*

**Keywords:** *Asparagus racemosus*, Galactogogue, Clinical trial, Ayurvedic medicine

## Abstract

*Asparagus racemosus *Willd. has repeatedly been mentioned as a galactogogue in Ayurvedic literature and has been confirmed through animal experiments as well. This randomized double-blind clinical trial evaluates its galactogogue effect in 60 lactating mothers by measurement of changes in their prolactin hormone level during the study. Several secondary parameters namely mothers’ weight, babies’ weight, subjective satisfaction of mothers and well-being and happiness of babies were studied to corroborate the primary findings. The oral administration of the research drug led to more than three-fold increase in the prolactin hormone level of the subjects in the research group as compared to the control group. The primary findings were corroborated by the secondary outcome measures and were found to be statistically significant (p < 0.05).

## Introduction

*Asparagus racemosus *Willd. (Liliaceae family) is a tall, climbing, thorny undershrub with triquetrous spiny branches and white flowers which is found in tropical and sub-tropical regions of india, australia and africa. It contains a large number of fleshy tuberous fasciculate roots which are soft, pliable and light pale to brown in color (1, 2). It has been prominently mentioned in Ayurvedic literature due the diuretic, antidysenteric, nutritive, galactogogue, aphrodisiac and antispasmodic properties of its roots (3). Its role as a milk enhancing substance or galactogogue, has been mentioned in several ancient Ayurvedic text books such as *Charak samhita *and *Susruta samhita *(4, 5). 

The chemical analysis of the roots of *A. racemosus *Willd. with different solvents has revealed the absence of flavonoids and the presence of several pharmacologically active saponins such as shatavarins I-V (antioxytocic, immunomodulator), sarasasapogenin, steroidal saponins (antioxytocic), immunoside (immunomodulator), anthocynanin (immunomodulator, antioxidant, blood cholesterol reducer), cyanidin glycosides (anti-protozoal), phytoecdysteroids (antimicrobial), glycoside-AR-4 (anti-protozoal), asparagamine A (antioxytocic, antitumor), racemofuran (antioxidant) and diosgenin (hepato-protector, antitumor) (6-8).

Mother’s milk is the most suitable nutrient for the baby. During the first six months of life, the breast milk is the optimal source of nutrition for babies. It is comprised of nutrient proteins, non-protein nitrogen compounds, enzymes, lipids, oligosaccharides, hormones, growth factors, host defense agents, vitamins A, C, B complex, binding proteins, lysozyme and antibodies, as well as many other factors that build a strong and healthy human being. The immunoprotective elements of breast milk include secretory IgA, lactoferrin, lysozyme, oligosaccharides, milk lipids and milk leukocytes. However, the secretion of milk from the mammary glands is controlled to a great extent by the concentration of prolactin hormone, a high molecular weight protein hormone secreted by the anterior pituitary gland, in the mother’s blood vessels. There is enough evidence now to support the belief that the prolactin hormone is directly responsible for promotion of milk secretion and that abnormally low levels of this hormone is indicative of deficient lactation in mothers (9). 

Oral administration of roots of *Asparagus racemosus *Willd. increased the milk yield in rats, cows, buffaloes (10-13) and goats (14), The crude alcoholic extract of the roots increased the weight of mammary glands in post-partum and oestrogen-primed rats and the uterine weight in oestrogen-primed group (11). However, there were no scientific evaluations based on clinical trials on lactating mothers about the galactogogue effect of this medicinal plant.

This research was aimed at the clinical evaluation for galactogogue efficacy of *Asparagus racemosus *Willd. roots by direct measurement of the prolactin hormone level and assessment of the secondary outcome measures.

## Experimental

This clinical trial was a double-blind, randomized, placebo-controlled, parallel-group study involving a total of 60 subjects. The study was carried out at the medical outdoor patients clinic department of the Institute of Post Graduate Ayurvedic Education and Research at the S. V. S. P. Hospital, Kolkata.

*Preparation of the research drug and its administration*

Fresh roots of *Asparagus racemosus *Willd. were collected from a reputed herb supplier of kolkata. These were authenticated and identified by the department of ethnobotany, botanical survey of india, shibpur, howrah. A voucher specimen (No. BM/UCM/007) was deposited in the herbarium before their utilization. The roots were cleaned, dried in shade and fine-powdered up to 80 mesh size. The root powder was put into capsules depending on the bodyweight of each subject and labeled “R”. Similarly placebo capsules were prepared with fine rice powder and labeled “C”.

*Acute oral toxicity*

Acute oral toxicity refers to the adverse effects that occur following oral administration of a single dose of a substance or multiple doses given within 24 h. OECD Guideline No. 425 (acute oral toxicity-acute toxic class method) was undertaken as a test procedure to ascertain the acute oral toxicity. In addition to the estimation of LD_50 _and confidence intervals, the test allows observing the signs of toxicity. Six groups each containing six swiss albino mice weighing 25-30 g maintained in standardized environmental conditions (animal house Reg. No. 1180/ac/08/CPCSEA) were fasted 12 h before the experiment and administered the drug sample orally in the dose of 300 mg/Kg, 500 mg/Kg, 1000 mg/Kg, 2000 mg/Kg and 5000 mg/Kg in 1 mL of aqueous solvent. The physical activity, behavior and mortality of each mice of each group is observed and recorded after 1/2 h, 2 h, 4 h, 8 h, 12 h, 24 h and 48 h. 

*Subjects*

All the subjects were lactating mothers who were selected after general examination from the medical outdoor patients department of the Institute of Post Graduate Ayurvedic Education and Research at the S. V. S. P. Hospital, Kolkata using the following inclusion criteria: (I) age of mother between 20-40 years; (II) age of infant up to 6 months; (III) having one or more of the following symptoms: deficient lactation, infant’s crying just after feeding, painful sensation in breasts during the time of feeding, loss of appetite in mother or the manifestation of any anxiety disorder which could effect the lactation.

*Ethical aspects*

All subjects were given verbal and written information about the potential risks and benefits of participation in the study. Written consent was required before randomization. The research followed the guidelines of the declaration of helsinki and tokyo for humans. The institutional clinical research committee of the participating center approved the study protocol.

*Treatment allocation and blinding*

Initially 70 subjects were selected for the purpose of this study, out of which 60 were finally followed up. These subjects were randomly allocated to one of the two equal-sized treatment groups and received the treatment dose of 60 mg Kg^-1^ body weight per day. The treatment groups included group R (research group) which received the root powder of research drug (60 mg Kg^-1^ body weight per day) and group C (control group) who were administered rice powder as placebo (60 mg Kg^-1^ body weight per day). The treatment consisted of oral ingestion of the treatment drug in the form of capsules three times daily with milk for 30 days. All the subjects were advised to immediately discontinue the use of the drug in case of any serious side effects.

The study medication was provided in white paper boxes, numbered consecutively with a medication number. The treatment allocation schedule was based on computer-generated random numbers. The treatment codes resided with the principal investigator and the local investigators were not aware of treatment assignments. No treatment code was broken before the last follow-up visit completion.

All the subjects were advised to lead their normal lives with their family during the study period subject to the following conditions: (I) no use of any contraceptive pills or steroid containing drugs which could affect the normal hormonal balance, (II) normal feeding technique and schedule for infants, (III) making sure of the babies’ burping after the feeding and (IV) avoiding any situation or events which could cause abnormal anxiety or tension. Follow-up visits were done on a weekly basis to undertake the physical examination of mother and child and for analysis of their symptoms.

*Outcome measures*

The primary outcome measure in this study was the biochemical estimation of prolactin hormone level before and after the treatment. To that end, a 2 mL sample of blood was drawn from each subject to ascertain the initial and final level of prolactin hormone level during the clinical study. The quantitative determination of prolactin hormone concentration (ng/mL) in human serum of blood sample was done by Micro plate Immunoenzymometric Assay (ELISA) technique at ashoka laboratory, kolkata using the kit of monobind. Inc. 

The secondary outcomes measured during this study were the changes in mothers’ weight, babies’ weight, subjective satisfaction of mother regarding the state of lactation and the well-being and happiness of babies. The mothers’ weight and babies’ weight were recorded just before and after the period of clinical trial. The subjective satisfaction of mothers regarding the state of lactation and the well-being and happiness of babies were rated on a graded scale ranging from 1 to 5 (1 denoting unsatisfactory and 5 representing highly satisfactory).

*Statistical analysis*

The results were analyzed statistically using the student’s t-test. The values of p < 0.05 were considered statistically significant, p < 0.01 were considered very significant and the values of p < 0.001 were taken as highly significant.

## Results and Discussion

*General information*

A total of 60 subjects were randomized and received trial medication after providing the written agreement of their participation in the trial. The mothers were of an average age of 25.6 years while the average age of infants was 2.8 months. 79% of the patients belonged to the minority muslim community, while 71% resided in an urban or semi-urban area. There was no significant group difference with regard to distribution of age, community or habitat. A total of 10 patients, who did not participate in the entire trial or did not turn up for regular follow-up visits, were excluded from the study.

*Acute oral toxicity*

No signs of oral toxicity were observed during the short-term analysis in mice but mortality was noticed in long-term study at the dose of 5000 mg/Kg.

*Evaluation of increase in prolactin hormone level*

The mean prolactin hormone level of the subjects showed a percentage increase of 32.87 ± 6.48 through the study period in case of the research group (group R) while the control group (group C) showed a percentage increase of only 9.56 ± 4.57 in the mean prolactin hormone level during the same period as detailed in Table 1.

**Table 1:** Mean percent increase in primary and secondary parameters during clinical trial

**Parameter**		**Mean percent increase**		
		**Group R**		**Group C**
**Primary outcome:**				
**Mean prolactin hormone level**		32.87 ± 6.48 ^a^	9.56 ± 4.57 ^a^	
**Secondary outcomes:**				
**Mean weight of mother**		3.78 ± 0.68 ^a^		1.37 ± 0.44 ^a^
**Mean weight of babies**		16.13 ± 3.65 ^a^		5.68 ± 2.57 ^a^
**Subjective satisfaction of mother**		1.54 ± 0.28 ^a^		0.48 ± 0.33 ^a^
**Overall well-being & happiness of babies**		1.27 ± 0.45 ^a^		0.29 ± 0.23 ^a^


n = 20          a: p < 0.05          b: p < 0.01          c: p < 0.001 The obtained results proved to be statistically significant (p < 0.05) using the 2-tailed t-test

*Evaluation of secondary outcome measures*

During the study period, the weight of the mothers showed a mean percentage increase of 3.78 ± 0.68 in case of group R, while the mean percentage increase was 1.37 ± 0.44 in case of group C. Similarly, the average percentage increase in the weight of babies was 16.13 ± 3.65 in case of group R and 5.68 ± 2.57 in case of group C during the treatment period.

The first secondary parameter, namely the subjective satisfaction of mothers regarding the state of lactation, showed an average increase of 1.54 ± 0.28 rating points in case of group R and 0.48 ± 0.33 rating points in case of group C during this period. The overall well being and happiness of babies, the other secondary outcome measure, was also rated by the mothers just before and after the study period. This parameter showed a mean increase of 1.27 ± 0.45 points in case of the research group and 0.29 ± 0.23 rating points in case of the control group as given in Table 1.

The results obtained for the secondary outcome measures were found to be statistically significant (p < 0.05).

Nature has intended to strengthen the bond between the mother and her infant by making mother’s milk a necessity for the baby. It has been proved that the breast milk is the best milk for the new born babies (15). The digestive system in a newborn baby is so delicate that it can easily digest only the breast milk. It is not only nutritious for the baby, but also helps protect him/her from almost all the infections by boosting his/her immunity level. The World Health Organization (WHO) and the American Academy of Pediatrics (AAP), both recommend exclusive breastfeeding for the first six months of life and then supplemented breastfeeding for at least one year and up to two years or more (16, 17). Exclusive breastfeeding for the first six months of life provides continuing protection against diarrhea and respiratory tract infections that are more common in babies formula-fed (18). The prolactin hormone produced in the anterior pituitary gland, promotes milk secretion in the mother. Higher levels of prolactin hormone in the lactating mother lead to promotion of milk secretion (6, 19). 

According to the Ayurvedic medicine, mother’s milk is the best nutritive substance for the child since it is sweet, emollient, laxative, wholesome, appetizing and easy to digest. It contains all the necessary vitamins and minerals which protect and nurture the baby. It originates from the breasts when the essence of digested food circulates all over the body and gets concentrated in the breasts of mother (4, 5). Deficient lactation can be caused by a number of factors such as anger, grief, lack of affection towards the child and *etc*. *Asparagus racemosus *Willd., has been described in many ancient Ayurvedic textbooks regarding its galactogogue action. The administration of alcoholic extract of its rhizome in adult pregnant female albino rats suggests an estrogenic effect of Shatavari on the female’s mammary gland and genital organs (20). The extract of Shatavari has been shown to increase both the weight of mammary lobulo-alveolar tissue and the milk yield in animal experiments. This effect was attributed to the action of released corticosteroids or an increase in prolactin. Shatavarins I-V, the steroidal saponins, may be responsible for the hormonal like effect of Shatavari and explain its traditional use as a reproductive tonic (21, 22). The presence of steroidal saponins and sapogenins constituents has been shown to directly contribute in the lactogenic effect of *Asparagus racemosus *(23, 24). Its galactogogue activity was evaluated experimentally on rats, cows, buffaloes and goats, but there were no similar findings regarding its galactogogue effects on human subjects (10-13).

The results of the clinical study showed that the oral administration of the research drug had a definite positive impact on the primary parameter, the prolactin hormone level in the lactating mothers. The increase in the mean prolactin hormone level of the subjects in the research group was three times more than that of the subjects of the control group (or more) during the study period. The findings in respect of the primary parameter are also corroborated by the results in case of the secondary outcome measures. Thus, while the measured secondary parameters, namely the weight of the mothers and the weight of the babies, showed substantially higher increases in the research group as compared to the control group, the rated secondary parameters, namely the subjective satisfaction of mother regarding the state of lactation and the well-being and happiness of babies, also showed a manifold increase in the overall ratings over the duration of the study in the research group subjects when compared to the control group. The research drug was also found to be quite safe from the viewpoint of acute oral toxicity. The statistical analysis of the results showed that the findings were statistically significant (p < 0.05).

## Conclusions

Evaluation of the galactogogue action of the roots of *Asparagus racemosus *Willd. during clinical trial on lactating mothers having symptoms of deficient lactation exhibits significant galactogogue activity in comparison with the control group without any significant acute toxicity effect. A probable reason for this galactogogue effect could be the presence of steroidal saponins in this plant. This drug has been scientifically validated for its galactogogue activity by using the modern parameters such as the prolactin hormone which is biochemically responsible for the lactation and also other associated symptoms. The overall research findings corroborate and validate the galactogogue activity of the research drug, which has been traditionally ascribed to it in the ancient text Charak Samhita.
